# Tactile spatial discrimination on the torso using vibrotactile and force stimulation

**DOI:** 10.1007/s00221-021-06181-x

**Published:** 2021-08-23

**Authors:** Atena Fadaei Jouybari, Matteo Franza, Oliver Alan Kannape, Masayuki Hara, Olaf Blanke

**Affiliations:** 1 Laboratory of Cognitive Neuroscience, Center for Neuroprosthetics, Faculty of Life Sciences, Swiss Federal Institute of Technology (EPFL), Geneva, Switzerland; 2 Laboratory of Cognitive Neuroscience, Brain Mind Institute, Faculty of Life Sciences, Swiss Federal Institute of Technology (EPFL), Geneva, Switzerland; 3 Graduate School of Science and Engineering, Saitama University, Saitama, Japan; 4 Bertarelli Chair in Cognitive Neuroprosthetics, Center for Neuroprosthetics and Brain Mind Institute, School of Life Sciences, Campus Biotech, Swiss Federal Institute of Technology (EPFL), 1012 Geneva, Switzerland

**Keywords:** Force vest, Vibrotactile vest, Tactile anisotropy, Tactile direction discrimination, Tactile localization, Torso-worn interface

## Abstract

There is a steadily growing number of mobile communication systems that provide spatially encoded tactile information to the humans’ torso. However, the increased use of such hands-off displays is currently not matched with or supported by systematic perceptual characterization of tactile spatial discrimination on the torso. Furthermore, there are currently no data testing spatial discrimination for dynamic force stimuli applied to the torso. In the present study, we measured tactile point localization (LOC) and tactile direction discrimination (DIR) on the thoracic spine using two unisex torso-worn tactile vests realized with arrays of 3 × 3 vibrotactile or force feedback actuators. We aimed to, first, evaluate and compare the spatial discrimination of vibrotactile and force stimulations on the thoracic spine and, second, to investigate the relationship between the LOC and DIR results across stimulations. Thirty-four healthy participants performed both tasks with both vests. Tactile accuracies for vibrotactile and force stimulations were 60.7% and 54.6% for the LOC task; 71.0% and 67.7% for the DIR task, respectively. Performance correlated positively with both stimulations, although accuracies were higher for the vibrotactile than for the force stimulation across tasks, arguably due to specific properties of vibrotactile stimulations. We observed comparable directional anisotropies in the LOC results for both stimulations; however, anisotropies in the DIR task were only observed with vibrotactile stimulations. We discuss our findings with respect to tactile perception research as well as their implications for the design of high-resolution torso-mounted tactile displays for spatial cueing.

**Supplementary Information:**

The online version contains supplementary material available at 10.1007/s00221-021-06181-x.

## Introduction

As the human torso provides an extensive skin area to convey tactile information, torso-worn haptic displays deploying tactile spatial cues have gained increasing attention in recent years (Rupert 2000; Lemmens et al. 2009; Arafsha et al. 2015; Lentini et al. 2016; Wacker et al. 2016; Buimer et al. 2018; Garcia-Valle et al. 2018). Moreover, while providing tactile information on the torso, a person’s active body parts, such as hands and fingers, remain fully available for daily living activities. Accordingly, the torso has been considered as one of the most available and practical candidate sites for wearable and mobile tactile communication systems (Cholewiak et al. 2004; Kristjánsson et al. 2016) and may also be particularly suited for applications in cognitive and clinical neurosciences (Rognini and Blanke 2016). However, to effectively convey spatially encoded tactile information and make use of this information, more data about the tactile spatial discrimination of the torso are required. Considering applications of torso-based tactile displays that provide spatially encoded tactile information, two general aspects of the tactile spatial discrimination are often tested: how well can a user spatially localize contacts on the torso? How well can a user distinguish tactile cues applied to neighboring locations on the torso? The former is referred to as tactile point localization and the later as tactile spatial acuity. Research on tactile spatial resolution was launched in the nineteenth century by Ernst Heinrich Weber (1834) and much later investigated by Weinstein (1968) and Stevens and Patterson (1995). Since then, however, only a handful of studies have investigated tactile perception on the torso (discussed below). In the present study, we describe new haptic touch systems and focus our investigation on assessing tactile spatial discrimination of the human torso.

Tactile point localization (LOC), evaluates a person’s ability to localize the point of tactile stimulation in an array of stimulators mounted on the torso. Previous studies that used a linear one-dimensional array of vibrators around the waist reported LOC accuracies within the range of 74% (12-tactor with 72 mm spacing) to 98% (8-tactor with 107 mm spacing). It was noted that accuracy tended to increase by increasing inter-stimulator spacing and at locations closer to the body midline (i.e., navel and spine) (Cholewiak et al. 2004). The higher localization ability in proximity to specific anatomical reference points, such as the body midline (e.g., navel and spine) and joints (e.g., wrist), was first described by Weber (1834) and was recently confirmed for the localization of both vibrotactile and static pressure stimuli presented on the upper limbs (e.g., wrist) (Cholewiak et al. 2004; Oakley et al. 2005; Cipriani et al. 2012). However, studies employing two-dimensional arrays of vibrators (e.g., 4 × 4 array) have not found higher LOC accuracy for midline regions and rather observed that LOC accuracy changes depending on the location of the target within the array (Lindeman and Yanagida 2003; Cholewiak and McGrath 2005; Jones and Ray 2008). For instance, accuracy was found to vary strongly (from 40 to 82%) depending on the position of the vibrator in the 4 × 4 array (Lindeman and Yanagida 2003; Cholewiak and McGrath 2005; Jones and Ray 2008).

Several other studies have investigated the spatial acuity for tactile cues applied to the torso and measured the capability of discriminating two nearby tactile stimuli presented on the skin surface. Although classical studies used the two-point discrimination test (Weber 1834; Weinstein 1968; Stevens and Choo 1996), more recent studies have questioned the validity of this measure as it is vulnerable to several possible confounds (e.g., two-point discrimination may be based on intensity rather than spatial cues). Alternatively, they suggested a task in which two successive stimuli are applied at nearby locations, and participants’ have to judge whether the two stimuli were delivered in the same location or not (Johnson and Phillips 1981; Johnson 1994; Stevens and Patterson 1995; Tong et al. 2013). For instance, Eskildsen et al. (1969) presented successive vibrotactile stimuli via a horizontal array of five vibrators on the back and reported a discrimination threshold at 10 mm on the back (at the level of the scapula). Van Erp (2005a) measured tactile direction discrimination (DIR) using two successive tactile stimuli on the torso, defined as the ability to discriminate whether a second tactile stimulus was to the left or to the right of a first tactile stimulus. They used a linear array of vibrators (11 in horizontal and 14 in the vertical direction). Using this method, they determined the tactile spatial acuity threshold at 20–30 mm on the torso, with better DIR accuracy (approximately 10 mm) only for horizontal array locations near to the body midline (i.e., navel and spine). They also highlighted the role of spatiotemporal factors by observing that the accuracy increased as the burst duration and/or inter-stimulus interval increased. The former effect is likely due to temporal summation of vibratory stimuli and reduced thresholds have been observed with increased duration of vibration (Gescheider et al. 2002). The latter effect may be caused by limitations in working memory and attentional resources in perceptual decision-making (Romo et al. 1999, 2002; Picard and Monnier 2009; Shah et al. 2019a). For instance, it has been shown that discrimination thresholds of two vibrotactile stimuli presented in different locations was systematically lower for sequentially vs. simultaneously presented cues; suggesting that if the time between two stimuli exceeds some minimum time required to process a single stimulus, the vibrotactile discrimination performance improves (Shah et al. 2019a). Finally, Jóhannesson et al. (2017) explored the impact of inter-stimulator distancing on tactile DIR accuracy [so-called relative spatial acuity; three-alternative force choice task (AFC)], using arrays of 3 × 3 vibrators on the lower thoracic region of the back. They reported that accuracy increased from 64 to 91% as an inter-stimulator spacing increase from 13 to 30 mm. Taken together, while LOC and DIR measure two different aspects of tactile spatial discrimination, previous studies often directly compared the results of these two tasks. To the best of our knowledge, there is no study investigating the degree of agreement or disagreement between the results of tactile localization and direction discrimination (as an indicator of tactile spatial acuity). Here, we investigate LOC and DIR on the torso in the same subjects, using 3 × 3 arrays of tactile stimulators.

The majority of torso-worn tactile displays developed in the last 2 decades have commonly adopted miniature affordable vibrotactile stimulators in commercial and experimental frameworks (Arafsha et al. 2015; Karafotias et al. 2017; Garcia-Valle et al. 2018). For instance, Van Erp and colleagues have employed torso-worn vibrotactile displays for use as a pedestrian navigation system (van Erp et al. 2003, 2005, 2005b, 2007). Lemmens et al. (2009) developed a wearable vibrotactile jacket to investigate the potential intensification of emotional immersion while participants watched a movie. Garcia-Valle et al. (2018) showed that using a haptic vest, which presented vibration patterns, improves immersion in multimodal virtual reality environments. Other studies have recently employed force stimulators to present collision-type touch stimuli (e.g., force, pressure, and compression) on the participants’ torso (Delazio et al. 2018; Al-Sada et al. 2019; Fadaei et al. 2021). For instance, a force jacket was made of pneumatically actuated airbags to provide strong and variable forces to the torso along with vibrotactile sensations (Delazio et al. 2018). In this line of research, it has been shown that the level of immersion in a virtual environment could be considerably enhanced by presenting ecologically valid touch feedback (Yoshikawa and Nagura 1997; Lopes et al. 2015; Cao et al. 2018). In addition, the processing of tactile spatial directional cues and notification has been described as more intuitive to participants when using force stimulation rather than vibrotactile as collision touch sensations are a more common haptic experience in daily life. Until now, however, research about tactile spatial discrimination on the torso has focused on performance for manually applied stimuli (Weber 1834; Weinstein 1968; Green 1982; Gibson and Craig 2005) and vibrotactile stimuli (Eskildsen et al. 1969; Jones et al. 2009; Hoffmann et al. 2018). To the best of our knowledge, there is no study investigating tactile spatial perception on the torso for directed force stimuli. Thus, it is not known whether force stimuli are characterized by improved tactile performance (compared to vibrotactile stimuli) in spatial discrimination tasks on the torso. Indeed, force and vibrotactile stimuli present some distinct features; for instance, vibrotactile stimuli are known to spread beyond the limits of the contact area (Cholewiak and Collins 2003; Sofia and Jones 2013; Shah et al. 2019b), while force stimuli are more focal, and this might lead to better accuracy in spatial discrimination. Here, we measured performance in LOC and DIR tasks and investigated the spatial accuracy of focal force and vibrotactile stimuli on the torso.

Moreover, previous studies have demonstrated directional anisotropies in tactile localization and tactile spatial acuity for both static pressure and vibrotactile stimuli. These studies reported higher tactile spatial performance along the transverse (limb) axis compared to the vertical axis. In particular, for the LOC task on the back, Jones and Ray (2008), using a 4 × 4 array of vibrators, observed that participants were better in the horizontal (87% correct) than vertical direction (68% correct) when using vibratory stimuli. In addition, for the DIR task, recently, Hoffmann et al. (2018) found that vibrotactile DIR accuracy is substantially higher in the horizontal axis compared to the vertical on the lower thoracic region, consistently across three different types of vibrators. Therefore, we also tested for any direction anisotropies in the LOC and DIR, using force and vibrational stimuli.

In summary, in the present study, we employed two body-conforming torso-based tactile displays (arrays of 3 × 3 vibrotactile stimulators: Vibrotactile vest; force stimulators: Force vest) and assessed tactile LOC and DIR on the skin surface of the human upper thoracic area. Using a within participant design, we (1) evaluated tactile spatial discrimination (LOC and DIR) of the upper torso region, (2) examined the association between the results of LOC and DIR tasks, and (3) compared performance when using vibrotactile and force stimulations. Finally, (4) we searched for directional anisotropy in both tasks and both types of stimulation.

## Materials and methods

### Participants

A total of 34 healthy participants (17 females, aged between 20 and 36 years, M = 26, SD = 4.2) were recruited for the experiment. All participants were right-handed [assessed via a 12-item Edinburgh Handedness Inventory (Oldfield et al. 1971)]. Pathological conditions affecting tactile sensitivity (e.g., skin alteration, chronic pain, and fractures) were excluded. They provided informed consent and ethical approval that was granted by the cantonal ethics committee in Geneva. All participants received a compensation of 20 CHF/h for their commitment to the experiment.

### Apparatus

#### Vibrotactile vest

**Fig. 1:**
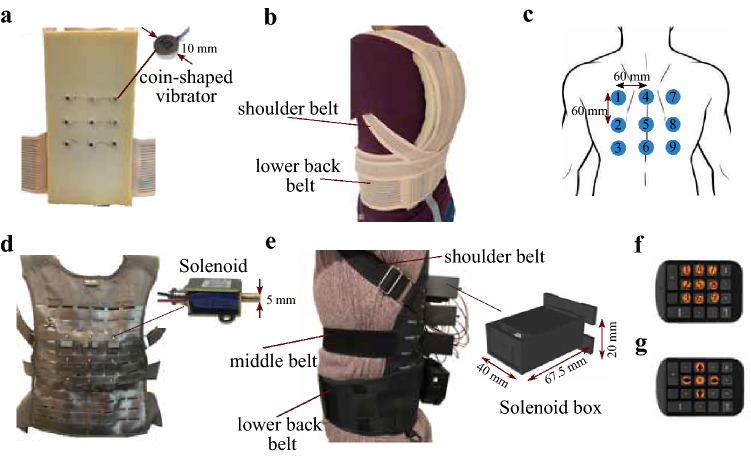
Experimental setup. **a** Interior view of the Vibrotactile vest with 3 × 3 of coin-shaped ERM vibrators. **b** The Vibrotactile vest on the participant. The vest was firmly fitted on a participants’ body with the lower back and shoulder belts. **c** Arrangement and numbering of stimulations for both Vibrotactile vest and Force vest. **d** Interior view of the Force vest with 3 × 3 push–pull solenoid actuators. **e** The Force vest on a participant. Three stretchable belts, including shoulder, chest, and lower back belts, firmly fixed the vest on the participants’ torso. Solenoids were placed in a custom-made 3D-printed box. **f** A numeric keypad with marked buttons was used to respond to the LOC task. **g** A numeric keypad with marked buttons is used to respond to the DIR task

The Vibrotactile vest consists of 9 (3 × 3) coin-shaped, Eccentric Rotating Mass (ERM) vibrators (310–003, Precision MicroDrive; body diameter: 10 mm; body length: 3.4 mm; weight: 1.1 gr) with an inter-tactor distance of 60 mm (Fig. 1a, c). The ERMs are controlled by haptic motor drivers (DRV2605, Texas Instruments) on 5 V (DC), resulting in a vibration frequency of 175 Hz and acceleration of 1.3 G. The haptic motor drivers were controlled with a microcontroller (STM32F407, STMicroelectronics; sampling time of 1 ms) which connects via Bluetooth to a host PC. A custom-made GUI was developed using the Qt platform (free and open-source platform to create GUI) to control vibrators and the experiment flows as well as record participants’ responses (i.e., entered via numeric keypad) along with the experiment. The ERM vibrators were attached to a 20-mm-thick foam (Softpur polyurethane foam) using glued-on snap fasteners. Vibrator foam was fixed to a fully elastic, posture-corrector brace using Velcro straps, allowing the experimenter to change or replace the vibrator foam easily. The Vibrotactile vest covers the whole back, and it is unisex. The front part of the brace includes elastic straps that wrap around the shoulder, chest, and lower back to ensure a snug and secure fit (see Fig. 1b). Moreover, the specific load frequency for the ERM vibrators was tested by activating each vibrator while the Vibrotactile vest was firmly fitted to a participant’s torso. The frequency of each vibrator was analyzed using real-time fast Fourier transform analysis (Audio Spectrum Analyzer dB RTA) on a mobile phone attached to the skin. Our test results showed that the load frequency ranged between 150 and 220, with an average of 175 Hz.

### Force vest

The Force vest is torso-worn and can apply focal force stimuli to the back. It was designed and prototyped in our previous study (previously named Cogno-vest) (Fadaei et al. 2021). It consists of nine (3 × 3) force stimulators (bi-directional, push–pull solenoid actuators; starting force: 5 N at 12 VDC, shaft length: 5.5 mm; shaft diameter: 5 mm; weight: 39 g), situated with an inter-stimulator distance of 60 mm on the back part of a tailor-made, Y-harness brace (Fig. 1d). To overcome gender-specific morphology, the front part of the brace consists of stretchable straps that wrap around the shoulder, chest, and lower back. The back part is made of polyester nylon with integrated laser-cut loops to support the hardware (Fig. 1e). Each force stimulator is embedded in a customized 3D-printed box, mounted on the back of the brace (see solenoid box in Fig. 1e). The Force vest is thus unisex and can keep stimulators flush against the skin. Arduino Mega 2560 controls the driving of solenoids via Bluetooth to a host PC with a sampling time of 1 ms (similar to the Vibrotactile vest). A custom-made GUI was implemented in the Qt platform to provide a convenient interface to control stimulators with the Force vest and to facilitate the running of the experiment. It also recorded participants’ responses (i.e., entered via numeric keypad). In our earlier study (Fadaei et al. 2021), the force stimulator (solenoid) performance was evaluated under various environmental and parametric conditions (e.g., the effects of the 3D-printed box, elastic tips, and stroke length). The results revealed that the realistic amount of force (average) provided by the Force vest is between 0.5 and 0.8 N depending on the stroke length (the force going up as the stroke length increases until 4 mm). The load frequency of the force stimulator was assessed and analyzed in the same way as described for the vibrotactile stimulator which indicates at which frequency the skin in contact with the solenoid would vibrate. The results revealed that the load frequency ranged between 500 and 1000 Hz, with an average of 650 Hz.

### Experimental design

Participants were exposed to a repeated measure design; participants wore both haptic vests (i.e., Vibrotactile vest and Force vest) and completed both the LOC and DIR tasks. In the experimental design for the LOC task, two factors were manipulated: stimulation type (vibrotactile vs. force) and stimulator location (9 locations on the upper thoracic region). For the DIR task, stimulation type (vibrotactile vs. force) and tactile orientation [three different orientation presentations, including horizontal (H), vertical (V), and double activation (DA)] were manipulated as independent within-participant variables.

### Procedure

**Fig. 2:**
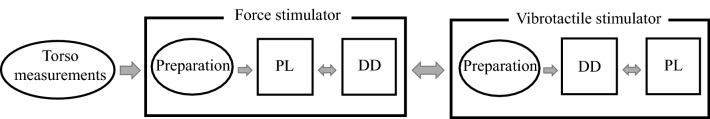
Experiment flow (simplified representation)

Participants were randomly assigned in a balanced way to the first experimental session with one of the two vests (Vibrotactile vest or Force vest). Subsequently, they performed both the LOC and DIR tasks in random order with a break (7 min). After, they changed the vest and were exposed to a second experimental session where they performed the same tasks in a counter-balanced order with respect to the previous session (see Fig. 2).

At the beginning of the study, participants were asked to wear a thin, fitted white T-shirt. This was done to eliminate any cloth-specific effect. Next, the experimenter performed the torso measurements, which included three measurements, namely torso length (vertical distance between the 7th cervical (C7 vertebra) and the top of the hip bone (iliac crest)), waist circumference (between the belly button and rib cage), and chest circumference (at the fullest part of the bust). Throughout the experiment, participants wore headphones playing white noise to conceal the activation noise generated by the haptic stimulators. The white noise intensity was customized for each participant to have full acoustic isolation.

Prior to each session, the experimenter helped participants to wear the haptic vests correctly. Stimulator arrays were placed centrally on the thoracic regions on the back, starting from the shoulder blades (scapula bones). Each session began with a calibration phase, where the experimenter activated each stimulator (i.e., vibrotactile or force) individually (with a duration of 250 ms and in a random sequence) to ensure that the participant could feel all stimuli by obtaining verbal confirmation. Due to the different nature of stimulators used in the Vibrotactile vest and Force vest, the calibration procedure was different for the two vests. For the Vibrotactile vest, in case of failure in perceiving the vibrotactile stimuli, the experimenter improved the perception by better fitting the vest on the participant’s torso. On the other hand, for the Force vest, the experimenter manually adjusted the force stimulator (solenoid) position at different stroke lengths until receiving verbal confirmation from the participant that they felt mechanical touch by each stimulator (more details can be found in (Fadaei et al. 2021)). The calibration tasks for the Vibrotactile and Force vest lasted approximately 5 and 10 min, respectively. Participants completed a training session, similar to the main task but shorter (around 1 min). During the training session, participants learned how to respond to tactile stimulation on their back with the corresponding keypad (see Fig. 2).

In the LOC task, a series of discrete tactile stimuli were applied to the participants’ back. They were instructed to indicate the location of the perceived stimulus, i.e., the position out of the total of 9 locations where the tactile cue had been applied (9 alternatives forced choice, 9-AFC). Participants responded by specifying a number that corresponded to the stimulated location by pressing a numeric keypad, as indicated in Fig. 1f). Participants did not receive performance feedback during the task. To reduce task complexity, they were asked to keep the keypad so they could see the buttons in the same order as the stimulators numbering on the back. In each trial, stimulators were activated for 250 ms with a random inter-trial interval of 2000 ± 250 ms. Tactile point activations (each of 9 different locations) were repeated 20 times, resulting in a total of 180 trials. To reduce potential fatigue, the task was divided into two blocks, each one including 90 trials and lasting 3 min. There was also a short break of around 2 min between two blocks.

In the DIR task, participants received two consecutive stimuli, and they were asked to determine whether the second was to the right, left, above, or below the first one or whether the same location was stimulated twice (5-AFC). Considering the arrangements of stimulators on the vests (3 × 3), shown in Fig. 1c, there are a possible 12 different horizontals (H; i.e., along with transverse axis) and 12 vertical presentations (V; i.e., along with longitudinal axis), and 9 DA of the same stimulator. Each of the orientational combinations was repeated 5 times, and the DA condition was repeated 6 times (to assess the same number of repetitions per condition), resulting in a total of 174 trials. The location of the first stimulus and the relative position of the second were randomly arranged. Participants responded via a standard numeric keypad with five marked buttons (see Fig. 1g) corresponding to the five possible response options, and the software recorded their responses. Similar to the LOC task, stimulators were turned on for 250 ms with an inter-stimulus interval of 50 ms. The inter-trial interval was altered randomly in the range of 2000 ± 250 ms. To avoid fatigue, the task was divided into two blocks of 87 trials, and each block lasted 4 min. There was a short break of approximately 2 min between two blocks.

### Statistical analysis

All analyses were performed in R (R Core Team 2020) running in the RStudio environment (RStudio Team 2020). In the DIR task, two participants had very low accuracy across the vests. Those data were excluded from DIR analysis as, presumably, the two participants did not understand the DIR procedure correctly. Thus, those analyses that involved DIR data only included data from 32 participants.

For the between-stimulus comparison, the overall accuracy (in percentage) of each task (i.e., accuracy for 180 trials in the LOC task and 174 trials in the DIR task) was considered as the response. A two-tailed paired sample *t* tests were used to assess whether accuracy differed significantly between vibrotactile and force stimulation. Cohen’s effect size was also reported to quantify the size of the difference between two groups. The chance level for LOC and DIR tasks were estimated at 11.11% and 20% since there were 9 and 5 possible response types in each trial, respectively. One-sample *t* tests were used to compare the accuracy with the chance level.

To better understand the LOC accuracy and its variation on the upper thoracic regions of the back, further analysis was conducted by considering accuracy (in percentage) at each location (accuracy of 20 trials presented at each location; 1–9) as the response. To investigate the participants’ ability to identify the stimulator’s location along with the vertical (column) and horizontal (row) axis on the back, data were collapsed across columns (upper, middle, and lower columns) and rows (right, middle, and left rows) stimulators. Thus, a linear mixed-effect model was performed to assess the effect of vibrotactile vs. force by considering stimulation rows, columns, and interactions between them as fixed effects and the participant as a random effect, accounting for between-subject variability.

To explore orientational biases in the LOC task, the number of localization errors was computed at each location for both vests. Mislocalization data were collapsed into adjacent (*N*_Adjacent_: confusion with stimulators that are situated one gap away from the target) versus nonadjacent biases (*N*_Nonadjacent_: confusion with stimulators that are situated at more than one gap away from the target), and horizontal (*N*_H_: number of errors made along with horizontal axis) versus vertical (*N*_V_: number of errors made along with longitudinal axis) biases. As inferred with the Shapiro–Wilk test of normality, mislocalization data significantly deviated from the normal distribution. Therefore, the Wilcoxon signed-rank test was employed to investigate the effect of adjacent and orientational biases.

To investigate the orientation-dependent effect in DIR results, mean accuracies for horizontal (DIR_H_; accuracy for 60 horizontal trials) and vertical (DIR_V_; accuracy for 60 vertical trials) trials were considered as the response. Then, a linear mixed-effect model was used by considering stimulation (vibrotactile vs. force), tactile orientation (H vs. V), and their interaction as fixed effects and subject as a random effect.

All post hoc comparisons were conducted using the Tukey HSD test. Pearson correlation coefficient was calculated to investigate the relationship between variables, and the *p* values were corrected for multiple comparisons (Bonferroni correction). In all analyses, significance was reported for *p* values smaller than 0.05.

## Results

### Overall accuracy

**Fig. 3:**
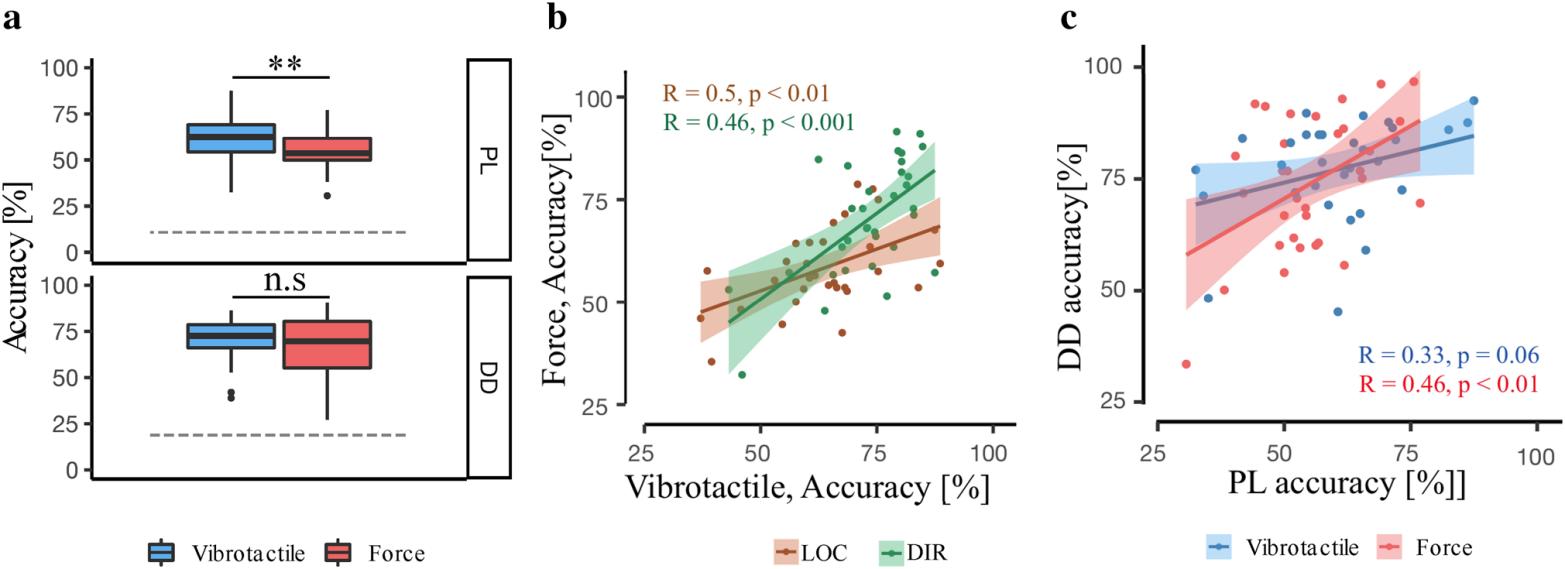
Overall accuracy results. **a** Box plot of overall accuracy for two stimuli across tasks. LOC accuracy was significantly higher with vibrotactile stimulation, while no difference was found between DIR accuracies of two stimulations. Gray dash-lines represents the chance level. Each box plot shows the median (50th percentile; dark bar), values to the 1.5 interquartile range (whiskers), 25th to 75th percentile range (box), and outliers (*p < 0.05 and **p < 0.01). **b** Scattered dot plot and Pearson correlation between accuracies with vibrotactile and force stimulations across tasks. There are positive correlations between the accuracies of two simulators for both tasks. Each point represents data from a single participant, and shaded areas show the 95% confidence interval for the regression line. **c** Scattered dot plot and Pearson correlation analysis between LOC and DIR accuracies of two stimulations. There is a significant positive correlation between LOC and DIR accuracies for force stimuli (in red) and not vibrotactile stimuli (in blue)

Overall accuracy results are represented in Fig. 3a. In both tasks, participants were significantly better than chance level [PL: vibrotactile: *t*(31) = 34.78, *p* < 0.001; force: *t*(31) = 29.9, *p* < 0.001; DIR: vibrotactile: *t*(31) = 25.77, *p* < 0.001; force: *t*(31) = 17.77, *p* < 0.001]. Performance in the LOC task was significantly higher [*t*(33) = − 2.92, *p* = 0.006; Cohen’s *d* = 2.85] with the vibrotactile stimulation (M = 60.70%, SEM = 2.32) versus the force stimulation (M = 54.6%, SEM = 1.95). For the DIR task, no significant accuracy difference was found in between the two stimulations [*t*(31) = − 1.54, *p* = 0.13; vibrotactile: M = 71%, SEM = 1.98%; force: M = 67.7%, SEM = 2.69%; Cohen’s *d* = 1.70].

The tactile performance was found to correlate between tasks (LOC, DIR) and stimulations (vibration, force). Thus, participants’ performance with vibrotactile stimulation significantly correlated with participants’ performance using force stimulation in the LOC task (*R* = 0.50, *p* = 0.002; brown line in Fig. 3b) and in the DIR task (*R* = 0.61, *p* < 0.001; green line in Fig. 3b). This was also found when comparing the two tasks, revealing significant correlations between the two tasks for force stimulation (*R* = 0.46, *p* = 0.007; red line in Fig. 3c), but for the vibrotactile stimulation, there was only a trend towards a significant correlation (*R* = 0.33, *p* = 0.060; blue line in Fig. 3c).

### LOC task

**Fig. 4:**
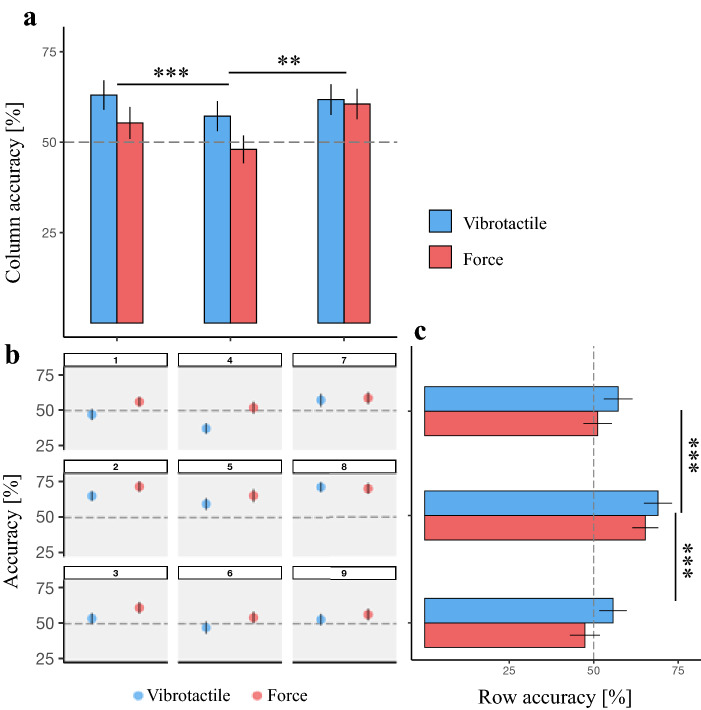
**a** Mean LOC accuracies at three columns in the array for both stimulations. **b** Mean LOC accuracies at nine stimulation landmarks in a 3 × 3 array for both stimulation types. **c** Mean LOC accuracies at three rows in the array for both stimulations. The dashed line shows the 50% threshold, and error bars illustrate the standard error of the mean (SEM) (*p < 0.05, **p < 0.01, ***p < 0 0.001)

The range of localization accuracies across both types of stimulation was 52.0–71.7% for vibrotactile and 37.5–65.1% for force stimulation, respectively. The mixed-model showed a significant main effect of stimulation [*F*(1, 573) = 11.22, *p* < 0.001], stimulation row [*F*(2, 573) = 28.52, *p* < 0.001], and stimulation column [*F*(2, 573) = 8.22, *p* < 0.001]; none of the interaction terms was significant. In line with the overall accuracy findings, participants’ LOC accuracy was significantly higher for the vibrotactile versus force stimulation [*t*(561) = − 3.34, *p* < 0.001]. Figure 4a shows that, whereas LOC performance in peripherial areas did not significantly differ [right-left columns: *t*(573) = 0.90, *p* = 0.6], performance was significantly more accurate for stimulations in peripheral than midline columns [right versus middle column: *t*(561) = 2.97, *p* = 0.009; left versus middle columns: *t*(561) = 3.87, *p* < 0.001, Fig. 4a]. In addition, as it is shown in the Fig. 4c, stimulations located in the middle row were more accurately perceived compared to the upper [*t*(573) = − 6.97, *p* < 0.001] and lower rows [*t* (66) = 5.79, *p* < 0.001]. No significant difference was found between accuracies of the upper and lower rows [*t*(573) = 1.18, *p* = 0.4].

Table 1 lists localization errors in the LOC task. For both stimulations, the majority of such errors were characterized by a mislocalization to an adjacent location (adjacent versus nonadjecent location: vibrotactile: *z* = 595, *p* < 0.001; force: *z* = 595, *p* < 0.001). Analyzing whether there was an axis along which localization errors predominated, we found that the number of horizontal errors was significantly lower than vertical errors, across stimulations (vibrotactile: *z* = 5, *p* < 0.001; force: *z* = 5.1, *p* < 0.001).

**Table 1:** Means of different tactile localization errors (standard error of the mean) for two vests

Error of localization	Force stimulation	Vibrotactile stimulation
*N*_Adjacent_	63.2 (2.91)	58.2 (3.21)
*N*_Nonadjacent_	1.38 (0.36)	0.91 (0.23)
*N*_H_	5.94 (1.1)	7.5 (1.34)
*N*_V_	53.6 (2.23)	47 (2.30)

We further compared the number of localization errors between the two types of stimulation for different categories of error (i.e., adjacent vs. nonadjacent, and H vs. V). Results showed no significant difference in the number of localization errors between the two stimulations (all *p* > 0.05).

### DIR task

**Fig. 5:**
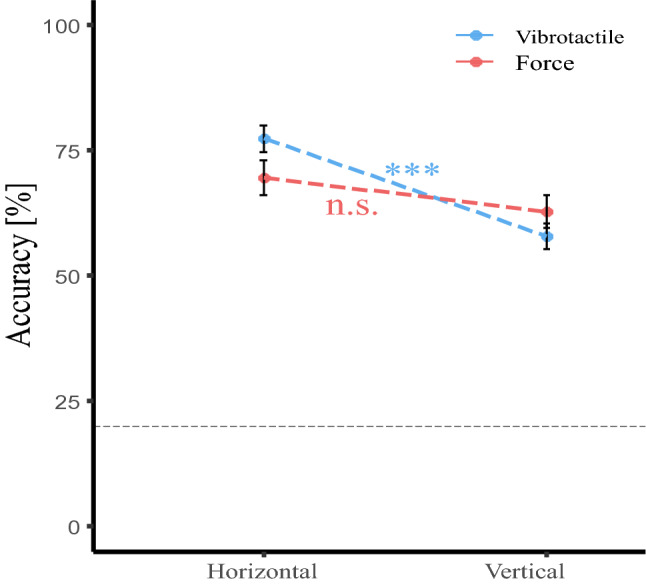
DIR accuracies with vibrotactile and force stimulations. The dash-line shows the chance level of 20%. The error bars show the standard error of the mean (SEM) (***p < 0 0.001; n.s.: No significant)

Investigating the effect of tactile orientation and of stimulation type on the accuracy in the DIR, we found a significant main effect for tactile orientation [*F*(1, 93) = 37.33, *p* < 0.001] and a significant interaction between the stimulation and tactile orientation [*F*(1,93) = 8.68, *p* < 0.001]. Post hoc analysis showed that participants were more accurate for trials presented along the horizontal axis only when using vibrotactile stimulation [*t*(93) = 6.76, *p* < 0.001; Fig. 5] (this effect was absent for force stimulation [*t*(93) = 2.24, *p* = 0.12)].

We assessed correlation coefficients separately for vertical and horizontal levels to explore any potential relationships between orientation-related effects in DIR accuracy (worse along the vertical axis) and localization errors in LOC (higher vertical errors). The only significant correlation was found for the vibrotactile stimulation in the vertical axis (DIR_V_-N_V_: *R* = − 0.36, *p* = 0.040; Bonferroni corrected *p* value), revealing that those participants who had higher vertical errors (*N*_V_) in the LOC task they also had low DIR performance in discriminating vibrotactile stimulation in vertical axis.

## Discussion

In the present study, we adapted two automatized tactile perception paradigms, namely tactile LOC and tactile DIR task, and measured tactile spatial discrimination on the human back. More specifically, we evaluated tactile perception over the thoracic region, using two custom-made haptic interfaces consisting of either vibrotactile or force stimulators. We found that LOC and DIR accuracy were slightly higher with vibrotactile stimulations than those with force stimulations. Using a within-participant design, we further demonstrate that tactile performance generalizes across tasks (LOC and DIR) for force stimulations but not for vibrotactile ones. Furthermore, we observed directional anisotropies in both tasks characterized by better performance for horizontal directions.

### Overall accuracy

We observed an overall LOC accuracy of 60.7% for vibrotactile and 54.6% for force stimulation in the thoracic region on the back. In the DIR task, we reported an overall accuracy of 71% for vibrotactile and 67% for force stimulation. Our LOC results (accuracy) are in line with earlier studies that employed a two-dimensional array of vibrators reporting LOC accuracy around 60% when stimuli were applied to the participants’ lower back (Cholewiak and McGrath 2005; Jones and Ray 2008). Considering the DIR task, our vibrotactile accuracy was lower than in a recent study (Jóhannesson et al. 2017), where the accuracy of 91% was found, even if the inter-stimulator distance of 30 mm was smaller than in our study (60 mm). However, the latter authors used a 3-AFC DIR task (via 3 × 3 vibrotactile array), which has a lower degree of difficulty compared to the 5-AFC used in the current study.

### Vibrotactile vs. force stimulation: correlation and comparison

We observed a moderate, positive correlation between performance for both stimulators across tasks (see Fig. 3c), suggesting that the spatial discrimination processes for both stimulation modalities, as tested in the present study, rely on similar perceptual mechanisms. Humans perceive tactile sensations (e.g., vibration, static pressure, and dynamic pressure) via particular sensory end organs, known as mechanoreceptors in the skin. It has been found that the sensitivity of mechanoreceptors relies on their receptive field size, density (i.e., many receptors in a given area resulting in high spatial acuity), frequency range, and the type of stimulation. Moreover, different body parts may contain various combinations of specific receptors, leading to varying perceptual capabilities in different regions (Hale and Stanney 2004; Choi and Kuchenbecker 2012). Load frequencies of the vibrator (175 Hz) and force stimulator (650 Hz) fall in the frequency range of the Pacinian corpuscle sensitivity (100–1000 Hz) (Vallbo et al. 1984) suggesting that both stimulation mainly activate Pacinian corpuscle receptors in the skin. The relatively low tactile spatial discrimination rate (around 60%) reported for both stimulations and across tasks further confirms the involvement of this type of subcutaneous mechanoreceptor, with its comparatively large receptive fields (Johnson and Yoshioka 2002). In comparison, non-Pacinian receptors embedded in the glabrous skin of the palm or fingers include greater density of receptors (Gregg 1951; Wilska 1954; Vallbo et al. 1995; Morioka et al. 2008) resulting in higher tactile spatial sensitivities (Weinstein 1968; Van Boven and Johnson 1994; Chen et al. 1995; Won et al. 2017).

While accuracy was higher for the vibrotactile than for the force stimulation across both tasks (mean difference, LOC: 6.1%; DIR: 3.3%), this difference was only significant for the LOC task. We fixed the spatial parameters (i.e., body site, inter-stimulator distance) and temporal parameters (i.e., burst duration and inter-stimulus interval) between the two stimulations as they may have a profound effect on both LOC and DIR results (Cholewiak et al. 2004; Van Erp 2005a; Jóhannesson et al. 2017). Nonetheless, we observed greater accuracy when using vibrotactile stimulation, suggesting that other stimulus-properties account for this discrepancy. These could include physical features (frequency, intensity, mass), contact area, the direction of movement with respect to the skin, and the amount of surface wave created by activating the motor. We here observed a higher accuracy with vibrotactile stimulations, which had a lighter weight (1.1 gr vs. 39 gr), slightly lower acceleration (1.3 G vs. 1.65 G), and lower load frequency (175 Hz vs. 650 Hz). Considering that the combination of mass, acceleration, and frequency of the tactile stimulation contributes to the perceived force, our results corroborate previous studies (Gibson and Craig 2006; Hoffmann et al. 2018), suggesting that the effect of physical parameters is not sufficient to account for the performance in the LOC and DIR tasks. With respect to the effect of frequency, previous studies on perceptual thresholds (e.g., detection threshold) have demonstrated that maximum sensitivity occurred at 220 Hz for hairy skin (with an inverse parabolic relationship) (Ribot-Ciscar et al. 1989; Cholewiak et al. 2004; Mahns et al. 2006; Jones and Sarter 2008). However, increases in frequency above 80 Hz (i.e., Pacinian corpuscle) have not been observed to improved tactile spatial discrimination performance (Cholewiak et al. 2004; Cholewiak and McGrath 2005; Hoffmann et al. 2018). Another possible explanation for the higher LOC and DIR accuracy with vibrotactile stimulations might also be that the contact area of the vibrotactile stimulator was twice as large as the one chosen for the force stimulator (vibrotactile: 314 mm^2^, force: 78.5 mm^2^). While the effect of the contact area on the LOC/DIR has not been systematically investigated, it would seem that due to the spatial summation of afferent signals from Pacinian corpuscles, vibrotactile thresholds (above 50 Hz) decrease as the stimulator area increases (Verrillo 1963; Gescheider et al. 2010), presumably enhancing the perceptual capabilities. In addition, we here observed that LOC and DIR accuracy were higher with the vibrotactile stimulator (coin-shaped vibrators), which generates motions parallel to the skin’s plane, compared to force stimulators that generate force perpendicular to the skin. This observation may relate to previous findings of Hoffmann et al. (2018), who found that DIR accuracy was higher with vibrators that generate motion parallel to the skin (as in our study) compared to the perpendicular to the skin as it provides stronger surface waves traveling on the skin (see Hoffmann et al. 2018 for more details). Future work is needed to investigate how tactile perception on the back depends on these different mechanisms.

### Association between LOC and DIR results

In the present study, we found a positive correlation between the LOC and DIR performances for the force stimulations but not for the vibrotactile stimulations. Although prior studies often directly compared the results of tactile localization (LOC) with tactile spatial acuity (e.g., DIR), our results suggest that extending the results of LOC to DIR (or the other way around) depends on the type of tactile stimuli and may not hold for vibrotactile stimulation (at least in the present study). The absent correlation between the LOC and DIR tasks with vibrotactile stimulations suggests that distinct and, presumably, stimulation-related driving factors are involved in the discrimination process of the vibrotactile DIR task as tested by us. As discussed below, the DIR task with the vibrotactile stimulation may more heavily depend on and vary with the viscoelastic properties of the participants' skin.

### LOC task

We observed that the LOC accuracies for both stimulations vary over the skin surface of the back in the thoracic region (vibrotactile: 52–71.68%, force: 37.47–65.08%). This observation may partly be explained by garment conformity but may also reflect differences in mechanoreceptor density on the torso. The present LOC performance is in line with previous studies that reported variation in tactile LOC (for both static pressure and vibrotactile stimuli) across the skin surface for different body parts, also including the back (e.g., forearm, abdomen, lower back, palm, and thigh) (Cholewiak and Collins 2003; Oakley et al. 2005).

### Lower LOC accuracy on the spine compared to the peripheral area

Concerning the LOC performance on the spine, previous studies have yielded mixed results and further depended on the physical arrangement of stimulators. Some studies that used a one-dimensional array of vibrators supported the enhancement of LOC in midline regions (close to the spine) as an anatomical body reference (i.e., as being related to the joints of the body) (Boring 1942; Cholewiak and Collins 2003; Cholewiak et al. 2004; Van Erp 2005a; Jones and Ray 2008). Others, however, did not report improved LOC in midline regions when using two-dimensional arrays (Lindeman and Yanagida 2003; Jones and Ray 2008). In the present study, we observed that the LOC accuracy was lower for stimulators in the midline area (column 2) than those located peripherally (columns 1 and 3). These results match the findings of previous multi-dimensional setups, suggesting that by adding dimension to the tactile display (e.g., two-dimensional array), no midline advantage is observed. Compatible with this account, earlier studies on the LOC for the forearm also found increased accuracy at the edges of the arm compared to those in the center (Oakley et al. 2005; Chen et al. 2008; Cipriani et al. 2012). Based on the present data of enhanced performance for lateral stimulations, we speculate that perceptual and attentional mechanisms related to lateralized stimulations may boost performance. However, more work is needed to specifically test this hypothesis and its comparison with enhanced midline performance. We also note that structural aspects such as the higher curvature in the spinal area compared to lateralized torso locations and the consequent poorer fit of the vest and stimulators around the midline may also play an important role.

### Directional anisotropy

In the present study, we observed directional anisotropy in LOC performance in both tasks, as participants made considerably fewer localization errors in the horizontal than vertical axes (see Table 1). Using a 4 × 4 array of vibrotactile on the back, Jones and Ray (2008) also showed that participants were less accurate in identifying the correct row of activation than the column. These observations are also in line with earlier LOC studies that reported systematic biases in the vertical axis on the skin surface of the palm, thigh (Sofia and Jones 2013), and arm (Oakley et al. 2005; Chen et al. 2008; Cipriani et al. 2012; Sofia and Jones 2013). In addition, we, here, found that the level of anisotropy in LOC performance was comparable between the two tasks, suggesting that comparable mechanisms are involved in the spatial discrimination process for both stimulations. One possible explanation, as discussed above, is that the torso’s lateral sides function as a perceptual reference point (as located to the endpoints of the stimulus range) affording higher accuracy in the horizontal direction. It has also been proposed that receptive fields of afferent fibers and/or neurons in the spinal cord and somatosensory cortex are oval-shaped and elongated along the longitudinal-vertical axis (Cody et al. 2008). Thus, one may speculate that stimulators in the horizontal axis would more likely activate separate adjacent mechanoreceptors, leading to fewer localization errors along with the horizontal axis. However, these argumentations do not seem to apply for our observation concerning DIR results as anisotropy was only seen in the results with vibrotactile stimulations: higher DIR accuracy for vibrotactile stimulation in the horizontal axis than vertical. Our observation for vibrotactile stimuli is consistent with the findings of Hoffmann et al. (2018), who recently found the superior DIR accuracy for vibrotactile stimulations presented horizontally on the lower thoracic region, across different types of vibrators. We argue that anisotropy in DIR results is mainly influenced by the amount of surface waves created by force and vibrotactile stimulations and how they spread on the skin. In contrast to focal force stimulation, vibrotactile stimuli spread beyond the contact area in the form of surface waves (Cholewiak and Collins 2003; Shah et al. 2019b). As the skin is a highly viscoelastic tissue, its mechanical properties highly impact the spread of the surface wave from the vibration source. Some direct human and animal evidence suggested that skin stiffness is anisotropic with higher stiffness along with the vertical axis than horizontal (Brown et al. 1975; Schady and Torebjӧrk 1983; Alloway et al. 1989). Therefore, surface wave from a vibrating source may propagate further along with the vertical axis and hence excite mechanoreceptors some distance from the cite of the stimulation, which makes difficult the recognition of the stimulation direction. (e.g., in our case, up or down).

### Wearability challenge of torso interfaces

While waist-worn tactile displays have been widely used in many recent studies (Jones and Ray 2008; McDaniel et al. 2008) to investigate vibrotactile spatial discrimination (LOC and DIR) at the lower torso, very little research has assessed the tactile spatial resolution of the upper thoracic torso. One complication has been the large morphological differences in the human torso area (within and between-participants’ variability). Hence, forming and fitting the torso-worn haptic display to the participant’s body is a challenge in assuring consistent stimulus application (Mortimer and Elliott 2017). In our previous study (Jouybari et al. 2019), in spite of using gender-specific chest-belts, containing 3 × 2 vibrators (horizontal spacing of 15 cm and vertical spacing of 8 cm), we obtained poor localization accuracy of 30.7% (on average) stemming in the impaired interface design. We noted that the ideal interface for the upper torso should be sufficiently adjustable to guaranty correct fit, firm support, and free-breathing for users even during movements despite the huge morphological variations in the torso area. Therefore, we, here, developed and employed two unisex, body-conforming, torso-based tactile displays. We further provide quantitative and practical information to designers of torso-based tactile interfaces.

### Study limitations

The present study has several limitations. Although we evaluated and compared tactile spatial discrimination using dynamic force (push–pull solenoid) and vibrotactile (coin-shaped ERM) stimulators, we only controlled for the temporal and spatial parameters between the two stimulators. However, there are other parameters that we did not control with the present systems, such as physical parameters, contact area, or spread of vibration waves. Therefore, the generalization of these results to other setups have to be taken with caution. Future experimental investigations are needed to systematically investigate the effect of such individual actuator properties on spatial discrimination, which was beyond the scope of the current study. Second, the design and control of force simulators are more problematic than vibrotactile stimulators. Thus, force stimulators require actual contact with the human skin to be perceived; moreover, as reported in our previous study with a force vest (Fadaei et al. 2021), the solenoid’s impact force might change in the range of 0.5–0.8 N, depending on the stroke length. Such effects could lessen the quality of force perception resulting in lower tactile perception with force stimulators. Future work may monitor the uniformity of the perceived intensity across the array of actuators by employing an objective calibration procedure, where the participants are asked to rate the intensity of each individual actuators. Finally, for the field to advance, the same experimental procedures and tasks have to be applied across participants, conditions, and different research groups, further empowered by the application of psychophysical methodology.

## Conclusion

Collectively, although previous studies investigated the spatial discrimination of vibration stimuli on the back, our study is the first to investigate both localization and tactile direction discrimination of the upper thoracic spine for two different types of dynamic mechanical stimulations (vibrotactile and force) in a large group of healthy participants. Our findings suggest that designers can use force stimulators to design the torso-worn tactile interface to provide more ecological touch feedback with the (almost) similar level of tactile spatial discrimination accuracy as observed in widespread vibrotactile interfaces. We also noted that overall accuracy with both stimulations is still relatively low (around 60%), indicating that further technological improvements are required to improve torso-based tactile communication systems. Apart from technological advancement, we might speculate that long-lasting training might improve performance. Alternatively, we suggest taking advantage of multisensory-training protocols (i.e., a combination of tactile with auditory, visual, or vestibular) instead of unisensory protocols to produce greater and more efficient learning (Ghazanfar and Schroeder 2006; Shams and Seitz 2008; Proulx et al. 2012). Furthermore, our findings provide new insights into the association between the results of the LOC and DIR tasks on the torso, indicating that the generalization of the LOC results to DIR is only valid for focal force stimulations and not for vibrotactile ones which spread further away. These results suggest that studies using vibrotactile interfaces should ideally measure spatial discrimination with different measures in parallel to estimate the actual discrimination accuracy.

## Supplementary Information

Below is the link to the electronic supplementary material.

Supplementary file1 (DOCX 74 KB)

## Data Availability

The datasets generated during and/or analyzed during the current study are available from the corresponding author on reasonable request.

## Code availability

The datasets generated during and/or analyzed during the current study are available from the corresponding author on reasonable request.
