# Supplementary material for: Tactile spatial discrimination on the torso using vibrotactile and force stimulation
Source: Exp Brain Res. 2021 Aug 23;239(11):3175–88. doi: 10.1007/s00221-021-06181-x (PMC8541989; doi:10.1007/s00221-021-06181-x)
Supplement: Supplementary file 1 — Supplementary file1 (DOCX 74 KB) [file 221_2021_6181_MOESM1_ESM.docx]

**Tactile spatial discrimination on the torso using vibrotactile and force stimulation**

Atena Fadaei Jouybari^1,2^, Matteo Franza^1,2^, Oliver Alan Kannape^1,2^, Masayuki Hara^3^, Olaf Blanke^1,2^.

^1^ Laboratory of Cognitive Neuroscience, Center for Neuroprosthetics, Faculty of Life Sciences, Swiss Federal Institute of Technology (EPFL), Geneva, Switzerland

^2^ Laboratory of Cognitive Neuroscience, Brain Mind Institute, Faculty of Life Sciences, Swiss Federal Institute of Technology (EPFL), Geneva, Switzerland

^3^ Graduate School of Science and Engineering, Saitama University, Saitama, Japan

**^Corresponding Author:^**

Olaf Blanke

Bertarelli Chair in Cognitive Neuroprosthetics, Center for Neuroprosthetics & Brain Mind Institute,

School of Life Sciences, Campus Biotech, Swiss Federal Institute of Technology (EPFL), 1012

Geneva, Switzerland

E-mail: olaf.blanke@epfl.ch

Tel: +41 (0)21 693 69 21

Fax: +41 (0)21 693 69 22

Supplementary materials

Investigating the effect of gender and torso sizing on tactile spatial discrimination

Statistical analysis

We investigated the effects of the fitting and gender variabilities on the DIR and LOC performance results. Participants' torso sizings (i.e., torso length, waist circumference, and chest circumference) were measured before the experiment (explained in the procedure section). For the LOC task, the overall LOC accuracy was considered as the response. A linear mixed-effect model was used to assess the significant difference between the two stimulators by considering the stimulator type as a fixed factor, gender as a control variable, torso length, chest circumstances, and waist circumference as covariate and subject as a random effect.

For the DIR task, the overall DIR accuracy was considered as the response. A linear mixed-effect model was also used to assess the significant difference between the two stimulators by considering the stimulator type as a fixed effect, gender as a control variable, and torso length, chest circumstances, and waist circumference as covariate variables and subject as a random effect.

Results

Statistical analysis showed that the LOC accuracy was significantly higher with vibrotactile stimulators (F(1, 33) = 8.55, p < 0.01). Furthermore, we found a significant effect for the chest circumference (F(1, 29) = 6.98, p = 0.01); suggesting that the LOC accuracy improved by increasing the participants' chest circumference (see Fig. 1). This observation most likely further supports our argument that participants used torso edge as reference points with which stimuli can be associated. However, the effect of other covariant factors was insignificant (both p > 0.2). We also did not find any significant effect for the gender on the LOC results (F(1, 29) = 0.09, p = 0.77).

For the DIR task, statistical analysis revelaed no significant effect of the stimulator type (F(1,33) = 2.81, p =0.1). There was no significant effect for covarient factors (Torso length: F(1, 29) = 3.84, p = 0.06; waist circumference: F(1, 29) = 0.11, p = 0.73; chest : F(1, 29) = 2.85, p =0.1) neither for the gender factor (F(1, 29) = 0.12, p =0.73).

Vibrotactile

Force

Chest Circumference (cm)

LOC Accuracy [%]

**Fig. 5** LOC accuracy as a function of chest circumference (cm). Each point shows the average LOC accuracy of one participant with one of the vests.
